# Evaluating the strength of genetic results: Risks and responsibilities

**DOI:** 10.1371/journal.pgen.1008437

**Published:** 2019-10-11

**Authors:** Gregory S. Barsh, Gregory M. Cooper, Gregory P. Copenhaver, Giorgio Sirugo, Hua Tang, Scott M. Williams

**Affiliations:** 1 HudsonAlpha Institute for Biotechnology, Huntsville, Alabama, United States of America; 2 Department of Genetics, Stanford University School of Medicine, Stanford, California, United States of America; 3 Department of Biology and the Integrative Program for Biological and Genome Sciences, University of North Carolina at Chapel Hill, Chapel Hill, North Carolina, United States of America; 4 Department of Systems Pharmacology and Translational Therapeutics, University of Pennsylvania Perelman School of Medicine, Philadelphia, Pennsylvania, United States of America; 5 Department of Population and Quantitative Health Sciences, Case Western Reserve University, Cleveland, Ohio, United States of America

In this issue, we are publishing an Editorial Expression of Concern in connection with a recent article on the genetics of multiple sclerosis (MS) [1]. In brief, the authors used exome sequencing of families with multiple individuals diagnosed with MS to identify 21 missense or nonsense mutations in 12 genes, and they then suggest that these 12 genes provide a platform for additional research [2]. Following publication, concerns were raised about the validity of some of the statements made in the manuscript, leading us to a series of discussions, both internally and with the authors. The purpose of this editorial is to describe the sequence of events, the rationale for our eventual publication of the Editorial Expression of Concern, and, in doing so, comment and engender discussion more broadly on the role of scientists as editors in what can sometimes be a grey area: the causal relationship between genetic and phenotypic variation.

Shortly after the manuscript was published, it was brought to our attention that it was receiving negative attention on social media; several readers contacted us directly to express their concern about the accuracy of the conclusions. In response, we evaluated what had transpired during our peer-review, editorial and publication process. We found no evidence of scientific misconduct or conflicts of interest during the review or editorial process, and we uncovered no concerns about the integrity of the data itself. However, our attention was drawn to several discrepancies in the strength of the evidence supporting claims for causality for the variants discussed in the manuscript. The Discussion includes statements that,

*(1) “…it should be noted that replication of our findings is warranted as the extremely low MAF observed for these variants, and the relatively low number of carriers within families, precludes sufficient statistical power for meaningful linkage and association analysis”*; and

*(2) “…replication of our findings in additional multi-incident MS families is necessary to confirm a pathogenic role…”*.

In our opinion, those statements accurately and fairly represent the work. However, the manuscript also includes statements in the Abstract,

*(3) “…which nominated likely pathogenic variants for MS in 12 genes…”*, and the Author summary,

*(4) “…resulting in the identification of 12 rare genetic variants that are largely responsible for the onset of multiple sclerosis in these families”*.

In our opinion, those latter two statements are not justified by the results.

To some extent, the distinction between statement and overstatement of findings can be a grey area, and there is an argument to be made that the appropriate editorial response in this type of situation is passive: allowing, occasionally facilitating, self-correction of the literature through post-publication commentary, and/or considering manuscripts that describe replication or failures of replication in additional studies. In this situation, we decided that a passive response is insufficient because of the potential for harm. In particular, the term “likely pathogenic” is widely used in the medical genomics community to refer to a genetic variant that is supported by specific criteria and evidence that, overall, are thought to confer a very high degree of confidence of causing disease [3]. None of the 21 variants identified in the article meet ACMG/AMP criteria for being considered as “likely pathogenic” per this definition [3]. However, owing to the use of such language in the publication, those variants could be misinterpreted by patients, health care providers, diagnostic laboratories, or direct-to-consumer genetic testing companies as very likely to cause MS, which is not demonstrably the case.

We contacted the authors one week after publication to share our concerns, and to request that they consider issuing a correction of two sentences in the Abstract, two sentences in the Author summary, and one sentence in the Discussion. However, we were unable to reach an agreement, and because the nature of the corrections we deemed necessary to prevent harm would require author involvement, we decided to take independent editorial action.

Editorial Expressions of Concern can be used to alert readers when there are questions about the integrity of the data and the results, and we emphasize that is not the case here. We intend for this Expression of Concern to be an interim step in the course of a fuller evaluative process, and we have initiated an extensive examination of the situation, including our actions and decisions both pre- and post-publication. In particular, our failure to recognize the presence and potential impact of discrepancies in interpretation prior to publication represents a lapse in our editorial responsibilities, for which we apologize to the authors and the community.

Our attention to this situation was initiated by a communication on Twitter, which, by virtue of its immediacy and visibility, has become a popular mechanism for communicating scientific ideas, opinions and discoveries. However, it is important to emphasize that our concern and our actions are based on internal discussion and evaluation, and not on social media or social media metrics. A few hundred characters written quickly in response to a stimulus may be sufficient to communicate an idea but is rarely sufficient to evaluate one, and in this situation, we are motivated and driven by our responsibilities as a journal, publisher, and member of COPE.

We also wish to comment more generally on our role as *PLOS Genetics* editors in evaluating and publishing claims for the foundational building block of genetics: causal relationships between genotype and phenotype. For model organisms, causality can be demonstrated through phenotype output from mutational analyses. This offers a rigorous and uniform standard for reviewers and editors to evaluate claims of causality. For studies in humans, the situation is different, as the available means of generating proof are far more limited, and there exists considerable room for good-faith debate about both process and results. Indeed, some studies make use of multi-dimensional datasets, for which statistical methods development is an active area of research. Additionally, there is an argument to be made for avoiding type II as well as type I error [4–6], particularly when the overall goal is focused on genetic architecture or biological processes rather than gene discovery *per se*. For this reason, we believe that, in general, it is important not to define publication standards such that errors of any sort are considered intolerable; indeed, falsified hypotheses are an inevitable and essential product of the scientific method.

To be clear, we do not endorse a loosening of rigor for human genetic studies; to the contrary, as genome sequencing becomes more accessible, the risk of false positive results only increases, and we fully support using statistical frameworks for evaluating claims of causality, linkage, and association whenever possible [5]. Furthermore, when clinical, potentially life-altering decisions are being made, transparency, rigor, and precision of terminology are essential; these points are what motivate the current Expression of Concern [1]. At the same time, analysis of genotype-phenotype relationships are not an end, but a means to understand basic biology, and investigate pathophysiologic mechanisms that underlie human disease. Examples of the latter effort in which the potential impact of aggregate genotype-phenotype relationships may go beyond the identity of a specific causal variant include autism [7,8], Hirschsprung’s disease [9], and congenital heart disease [10]. Perhaps the same will be true of MS.
